# Exploring the potential of large language models in nutrition behavior prediction: evidence from college students

**DOI:** 10.3389/fnut.2026.1769064

**Published:** 2026-06-22

**Authors:** Misha M. Madhavan, Satyapriya, Subhashree Sahu, Praveen Koovalamkadu Velayudhan, Siva Smitha, Arjun Prasad Verma, Vikash Pawariya, Sukanya Barua, Girijesh Singh Mahra, Sitaram Bishnoi, Manjeet Singh Nain, Venu Lenin, Monika Wason, Rajarshi Roy Burman

**Affiliations:** 1 ICAR - Indian Agricultural Research Institute, New Delhi, India; 2 CoA, Vellayani, Kerala Agricultural University, Trivandrum, Kerala, India; 3 CoA, Banda University of Agriculture and Technology, Banda, UP, India; 4 CoA, Nagaur, Agriculture University, Jodhpur, Rajasthan, India; 5 Indian Council of Agricultural Research, New Delhi, India

**Keywords:** artificial intelligence, behavior prediction, behavioral nutrition, ChatGPT, healthy eating behavior, large language model, LLM, survey data

## Abstract

**Introduction:**

The integration of Artificial Intelligence (AI) and Large Language Models (LLMs) in behavioral nutrition is expanding rapidly to predict the behavioral data. Recent developments in LLM-assisted analytical tools have opened new possibilities for analyzing survey datasets and exploring behavioral patterns related to nutrition.

**Objectives:**

The study aims to evaluate the ability of a large language model-assisted analytical workflow to predict healthy eating behavior scores using survey data collected from undergraduate students. The study also compares the predictive performance of the LLM-assisted workflow with a baseline OLS regression model and examines how prompt-based conditioning using different dataset sizes influences prediction accuracy.

**Methods:**

This study employed a cross-sectional design using primary survey data collected from 914 undergraduate students from agricultural universities in India between December 2024 and February 2025. The Healthy Eating Behavior (HEB) scale was used to assess behavioral outcomes. The dataset was analyzed using an LLM-assisted analytical environment (ChatGPT-4o), where the model was prompted to generate predicted HEB scores across different training-test splits. The predicted scores were compared with observed survey scores using statistical tests. Additionally, qualitative analysis examined if the model’s predicted determinants of healthy eating behavior were aligned with the previous literature.

**Results:**

The results indicate that predictions generated using the LLM-assisted analytical workflow gradually converged toward the observed survey scores as the size of the training dataset increased. When a larger proportion of the data was used for training, the predicted scores did not differ significantly from the observed mean scores. However, predictive accuracy could be further strengthened using larger and more diverse datasets. The qualitative analysis also revealed similarity between the determinants of healthy eating behavior identified by the model and those reported in prior studies.

**Conclusion:**

The findings suggest that LLM-assisted analytical tools can support exploratory prediction tasks in behavioral nutrition research when sufficient training data are available.

## 1 Introduction

Large language models (LLMs), such as GPT-3 and GPT-4, are increasingly being explored for their potential to assist with analytical and reasoning tasks across diverse research domains. These models demonstrate strong capabilities in pattern recognition, language understanding, and statistical prediction across structured and unstructured data. Recent studies have examined whether LLMs can support more complex analytical tasks, including interpreting relationships between variables and generating plausible explanations from data (1, 2). However, despite these advances, current artificial intelligence systems primarily rely on statistical associations and probabilistic patterns rather than true causal inference, which remains as major challenge in AI research.

Although LLMs can generate explanations that resemble causal reasoning, their underlying mechanisms are largely based on patterns learned from large text corpora rather than explicit causal frameworks. They often reproduce causal language patterns (for example, statement like “high sugar intake leads to obesity”) without modelling underlying cause-and-effect structures. Several studies highlight these limitations, noting that LLMs struggle with causal discovery and structured causal inference without external models or domain knowledge (3–6). Zečević et al. (7) describe this phenomenon as “causal parroting.”

Despite these limitations, several studies have explored the potential of LLMs in reasoning-related tasks. Advanced models such as GPT-3.5 and GPT-4 show promising performance on selected structured reasoning benchmarks, including pairwise causal discovery, counterfactual reasoning, and event causality identification (8). LLMs can also generate counterfactual explanations and plausible responses when prompted appropriately (9, 10). However, performance remains uneven across tasks, with only moderate accuracy reported in some reasoning contexts (8). To address these limitations, researchers propose integrating LLMs with causal representation learning and external reasoning frameworks to enhance interpretation of complex relationships (11). In addition, recent evidence suggest that LLM-based systems can generate contextually appropriate behavioral responses in social situations, sometimes performing comparably to human participants in specific judgement tasks (12).

The integration of artificial intelligence (AI) and large language models (LLMs) into nutrition and food science research has expanded rapidly. These technologies are increasingly applied to analyze dietary data, support nutritional assessments, and assist in personalized dietary recommendations. AI-driven approaches can analyze large and complex datasets to identify dietary patterns and health outcomes, supporting personalized nutrition strategies (13, 14). Image recognition systems and mobile applications have also improved dietary monitoring and nutrient estimation (15). Beyond individual assessment, AI tools are used to analyze consumer preferences and anticipate emerging food trends (16).

Healthy eating behavior (HEB) is an important determinant of overall health and well-being. However, adoption of healthy dietary practices varies across individuals and populations due to cultural, socio-economic, and demographic influences. Understanding these differences is essential for designing effective dietary interventions. Previous studies show that demographic factors such as gender, socio-economic status, and cultural norms significantly influence eating behavior. For example, gender roles often shape food preparation responsibilities and dietary preferences (17, 18), while social norms influence associations between certain foods and gender identity (19).

In this context, recent studies have examined LLMs for forecasting and behavioral prediction tasks (20–22) and emerging evidence suggests that LLM-based systems may emulate aspects of human behavioral responses in experimental settings (23, 24). These developments raise the question whether LLM-assisted analytical workflows can be applied to behavioral survey data to predict health-related behaviors. Accordingly, this study examines whether a large language model (25) can assist in predicting healthy eating behavior by identifying statistical patterns in survey data collected from undergraduate students. The predictive performance of the LLM-assisted analytical workflow is compared with a conventional ordinary least squares (OLS) regression model as a baseline benchmark. The study further evaluates how training dataset size influences predictive performance in behavioral nutrition.

## 2 Related works

Lippert et al. (26) demonstrate that AI models, particularly GPT-4, can effectively predict behavioral science outcomes at scale and minimal cost. GPT-4 outperformed GPT − 3.5 in forecasting empirical findings related to emotions, gender, and social perceptions, with prediction-outcome correlation scores of 0.89 (GPT-4), 0.87 (human experts), and 0.07 (GPT − 3.5). The concept of Turing Experiments (TEs) was introduced to evaluate the ability of LLMs to simulate human behavior by replicating classic experiments, with recent models successfully reproducing findings from economic, psycho-linguistic, and social psychology studies (27).

AI tools have been used to extract, structure and analyze large-scale social media data to better understand dietary behaviors and public perceptions (28). The study by Chopra et al. (29) highlights the potential of LLMs in predictive modelling of complex behaviors such as eating disorders, demonstrating how analysing Reddit posts can help identify psychological stressors, coping mechanisms, and social influences. Such approaches may support earlier detection and more targeted behavioral interventions.

Advancements in AI-driven dietary assessment highlight the potential of technology in understanding eating behavior. Romero-Tapiador et al. (30) introduced AI4Food-NutritionFW, a framework for generating simulated food image datasets to support personalised nutrition recommendations. Kyritsis et al. (31) developed a smartwatch based monitoring system using neural networks to detect food intake events in real-time, thus enabling precise and personalised dietary interventions.

Despite these innovations, important challenges remain. Implementation of LLMs in behavioral science is constrained by data bias, misinformation risks, interoperability issues, limited interpretability, and ethical concerns (13–15, 32–34). Concerns regarding factual reliability, unpredictability, and limited contextual understanding further necessitate careful validation and interdisciplinary oversight (13, 14).

Recent studies have also applied natural language processing (NLP) techniques to large-scale dietary datasets. Choi et al. (35) employed topic modelling to identify dietary patterns from national nutrition survey data, and Choi et al. (36) integrated NLP-derived indicators with machine learning models to predict diet-related diseases. These studies highlight the value of combining NLP and predictive modelling in nutritional research. Researchers also examined how LLMs can generate synthetic user research data using prompt conditioning. It was reported that models like GPT-3 can produce realistic responses, making them useful for rapid, low-cost idea generation and pilot testing though results must still be validated with real data (37). The present study evaluates a large language model (LLM)-assisted analytical workflow using structured behavioral survey data, building upon earlier studies that established methodological foundations for computational approaches to dietary behavior analysis.

## 3 Methodology

In this section, we outline the approach and data used to evaluate the ability of a large language model to analyse behavioral survey data and generate predictions based on statistical associations between variables.

### 3.1 Behavior prediction using quantitative data

#### 3.1.1 Data collection

This study employed a cross-sectional design. Convenience sampling was used to select undergraduate students from agricultural universities across India. An online questionnaire was administered via Google forms and circulated through institutional WhatsApp groups. Data were collected between December 2024 to February 2025, yielding 914 valid responses. Respondents were enrolled in B. Sc. Agriculture, B. Sc. Community science and related programs. The dataset was cleaned, coded and prepared for comparative analysis using STATA, XLSTAT and an LLM-assisted analytical workflow.

#### 3.1.2 Variables selected for the study

This study evaluates the predictive ability of LLM-assisted regression analysis. The dependent variable was Healthy Eating Behavior (HEB), conceptualised as a multidimensional construct (38, 39). The Healthy Eating Behavior Scale developed by Liao et al. (38), was adapted to align with the Dietary Guidelines for Adults in India. The scale consisted 13 items, 8 assessing balanced dietary behavior, 1 regarding processed food consumption, 3 assessing food label use, and 1 assessing healthy food choice behavior. Responses were recorded on a 5-point Likert-type scale ranging from ‘never (0 days per week)’ to ‘always (7 days per week)’, scored from 1 to 5, with higher scores indicating healthier eating behavior. Nutrition literacy was included as a key predictor variable (38, 40, 41). The self-rated nutrition literacy scale developed by Liao et al. (38) was adapted for the study. The scale consists of five domains with eight items each, measured on a 4-point Likert-type scale (1 = very difficult to 4 = very easy), with higher scores indicating better nutrition literacy. Additional predictors of HEB were selected based on prior empirical literature (38, 39).

#### 3.1.3 LLM-assisted analytical workflow

The analysis was conducted using the LLM, ChatGPT (GPT-4o, OpenAI; accessed via the web interface, March 2025 version). Due to continuous model updates, results may vary slightly across versions. Further, default interface settings were used. As the experiments were conducted via the web interface rather than a version-controlled API, underlying model configurations may evolve over time, which may affect strict reproducibility.

Structured prompts were designed to instruct the LLM. The model enabled the execution of statistical computations through internally generated code. The cleaned dataset was uploaded in spreadsheet format and the model was prompted to perform regression analysis using specified dependent and independent variables. The outputs were exported and compared with results generated in STATA to verify consistency. For prediction experiments, the dataset was divided into training and test subsets. The training dataset included both predictor variables and the Healthy Eating Behavior (HEB) scores, while the test dataset included only predictor variables. The model was instructed through structured prompts to analyse statistical associations within the training data and generate predicted HEB scores for the test observations. Actual HEB scores for the test set were used solely for *post hoc* evaluation of prediction accuracy, thereby preventing data leakage.

This workflow represents an LLM-assisted analytical approach in which the language model acts as an interface for executing statistical analysis. The analysis relies on statistical associations observed in the training data rather than on formal causal inference methods. There have been many research evaluated LLMs’ causal reasoning abilities in several aspects including pairwide causal relation identification (8), commonsense causal reasoning (42), and graph-based tasks (43). Wang et al. (44), proposed a benchmark generation framework that can generate new, unseen causal questions to test the generalization ability of LLMs on causal inference on four tasks: causal path finding (CP), backdoor adjustment (BA), factual inference (FI) and counterfactual inference (CI). But in this study the model does not delineate causal relationships, quantify causal effects, or endorse intervention-based reasoning. Specifically, the LLM does not take into account confounding factors, does not use counterfactual reasoning, and does not use any formal framework for causal inference. The methodologies such as the Rubin causal model (RCM) and structural causal model (SCM) frameworks (45) necessitate explicit assumptions regarding treatment assignment, counterfactuals, and causal structure, which are absent in the current study. Consequently, its outputs are exclusively regarded as predictions derived from observed data patterns. This distinction has been clearly articulated to prevent any possible confusion between predictive modeling and causal explanation. Further, the LLM-based analytical workflow has several limitations, including sensitivity to prompt structure and phrasing, dependence on the provided data representation, and the absence of inherent statistical guarantees such as parameter estimation, confidence intervals, or convergence properties. The outputs depend on the input prompt and may vary across runs; therefore, the results should be interpreted with appropriate caution. Moreover, the LLM does not model underlying psychological, cognitive, or behavioral processes. Instead, it identifies and reproduces patterns present in the training data. Accordingly, the predicted HEB scores should be interpreted as pattern-based predictions rather than as evidence of human behavioral modeling or causal relationships.

#### 3.1.4 Prompt-based conditioning

**Figure 1:**
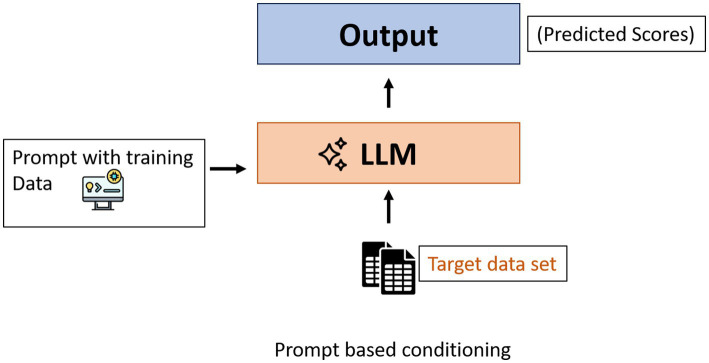
Conceptual illustration of prompt based conditioning. Adapted from (5), licensed under CC BY 4.0.

We employed prompt-based conditioning within the analytical environment rather than parameter-level fine-tuning (46). No model weights were updated; predictions were generated based on statistical patterns identified within the uploaded train dataset, using structured prompts as illustrated in Figure 1. In this study, training data set (train data set) refers to the prompt-conditioning dataset which provide structured contextual information to the model without retraining or fine-tuning of the model. The model parameters were not modified and that the uploaded dataset was used solely to inform the model’s predictions.

For prediction experiments, structured prompts was designed by explicitly defining the contextual role, variable descriptions, and causal relationships. The model was instructed to learn patterns from a training dataset and predict HEB scores for a test dataset, with outputs constrained to a 0–65 Likert scale in CSV format. The training dataset (predictor variables along with the corresponding HEB scores) and the test dataset (containing only predictor variables, with the HEB scores withheld) were uploaded as Excel files along with the prompt provided below.

“Carefully analyze the training dataset of 457 college students to understand how the [healthy_eating_behavior] scores are generated based on the given explanatory variables. After learning the underlying pattern or relationship, apply it for the given test dataset. Using the learned pattern, predict the [healthy_eating_behavior] score for each individual in the test dataset.

For each student, the columns in the training dataset can be described as follows: You are an undergraduate student studying at an agricultural university in India. Your parents’ education level is [parental_education] (ordinal level), your family’s annual income is [annual_income] and type of family is [family_type] (where 1 = joint family and 2 = nuclear family). You are currently staying at [place_stay] (where 1 = Rented accomodation outside campus, 2 = College hostel and 3 = with family at Home). You perceive your health status as [self_perc_health_status] where (1 = poor, 2 = fair and 3 = good). You get exposed to nutrition related information as [exposure_nutrition_info] where 0 = never, 1 = seldom, 2 = sometimes, 3 = often and 4 = always and [nutrition_courses] (where 0 = have not and 1 = have) undergone nutrition related courses. You perceive the need for nutrition related information as [self_perceived_need_nutrition_info] (where 0 = no need at all, 1 = somewhat needed, 2 = has a need and 3 = has a great need). Your nutrition literacy score is [nutrition_literacy] out of 32 in interval level. Consider all these variables as causes for your healthy eating behavior given by [healthy_eating_behavior] (Ranging from 0 to 65 measured using a likert type scale).

Format the output as a csv file with new coloumn added as [healthy_eating_behavior] (Ranging from 0 to 65 measured using a likert type scale).”

The same prompt was repeated for different prediction experiments by changing the sample size accordingly (Screen shots of the prompt used for different datasets and workflow are given in the Supplementary Information).

We also tested the ability of the ChatGPT to perform regression analysis and to identify the determinants of HEB by directly specifying these things through structured prompt The prompt specified the dependent variable, predictor variables, and required statistical procedures. The model was instructed to generate regression outputs and predictions in excel format to facilitate comparison with conventional statistical software STATA. The datasheet for performing the regression analysis was also uploaded along with the prompt.

#### 3.1.5 Prediction experiments using training and test datasets

The prediction experiments examined how training dataset size influences predictive performance. Prompt-based analysis using large language models differs from conventional machine learning pipelines and may introduce implicit dependencies due to sensitivity to prompt structure and interaction context. To address this, all experiments were conducted using standardized prompts, with strict separation of training and test datasets to avoid data leakage. Each experiment was performed independently, using separate sessions to prevent carry-over effects across different data splits.

In Experiment 1, the model generated predictions using the full dataset (N = 914) without applying a separate hold-out test set, serving as a baseline condition. In subsequent experiments (Experiments 2–5), the full sample was systematically divided into training and test datasets using progressively increasing training proportions of 50:50 (*n* = 457:457), 70:30 (*n* = 640:274), 80:20 (*n* = 731:183), and 90:10 (*n* = 823:91). In Experiment 6, a fixed training dataset of 800 observations was used, with the remaining observations allocated to the test set. This experiment was conducted to assess model consistency at a training size between the 80 and 90% split conditions. In each case, the predicted HEB scores were compared with the observed values to evaluate model performance. To ensure reproducibility, the dataset splitting procedures, prompt structure, and analytical workflow were kept consistent across experiments, with training dataset size as the only varying factor.

Prediction accuracy was evaluated by testing the null hypothesis:

*H*_0_: The mean difference between predicted and actual HEB scores equals 0.

*H*_1_: The mean difference is not equal to 0.

Normality assumptions were assessed prior to conducting paired t-tests to determine whether statistically significant differences existed between predicted and observed scores.

### 3.2 Identifying determinants of healthy eating behavior using qualitative data

Qualitative behavioral explanations often describe relationships among actions, motivations, and outcomes (47). Rigorous and trustworthy qualitative analysis is characterized by systematic, organized, and iterative procedures (48). Such approaches generate rich, in-depth insights that illuminate underlying mechanisms and processes (49, 50). However, qualitative research has faced criticism regarding replicability and methodological rigor. In response, scholars have developed a range of evaluative criteria and methodological techniques to strengthen analytic credibility and transparency (51, 52).

**Figure 2:**
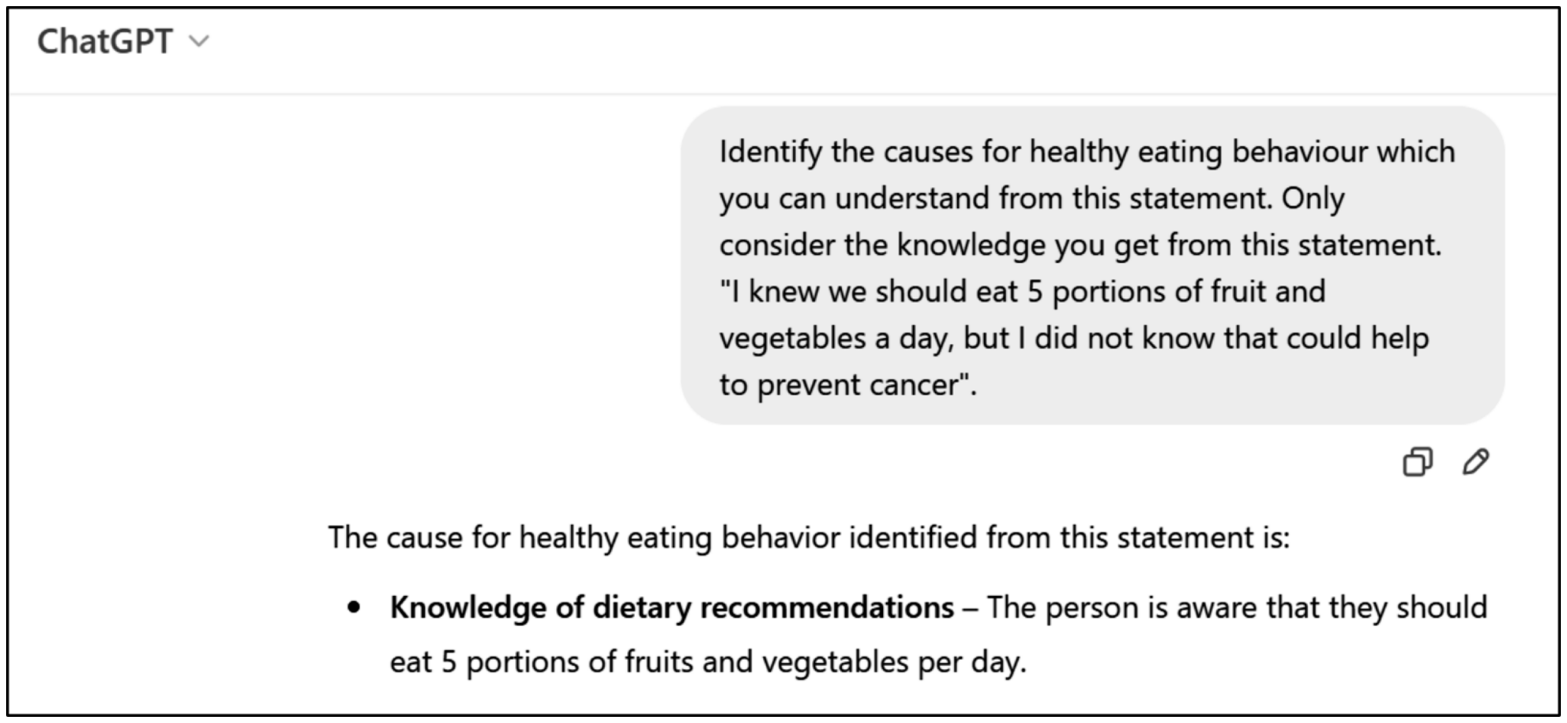
Prompt used for identifying behavioral factors from qualitative text.

In this study, selected qualitative statements related to healthy eating behavior were adopted from Zorbas et al. (39) and provided to the LLM (ChatGPT-4o) through structured prompts (Figure 2). The model was asked to identify behavioral factors reflected in the text and were systematically compared with those reported in the original study. Conceptual similarity was assessed and categorized as exact match, more specific and additional. “Exact match” refers when the LLM code and literature parent code represent essentially the same conceptual construct with equivalent meaning. “More specific” category indicates that the LLM code refines features within the broader parent category. “Additional” category represents that the LLM code represents a new dimension or subcomponent not explicitly captured in the original literature code. This mirrors flexible pattern matching logic: comparing observed patterns with theory-derived categories and systematically identifying alignment, divergence, elaboration, and novelty (53–59). This comparative framework enabled assessment of the LLM’s capacity to interpret behavioral factors from qualitative narratives.

#### 3.2.1 Hybrid thematic analysis

Hybrid thematic analysis combines inductive and deductive approaches to leverage the strengths of both. It involves using pre-defined categories based on theoretical frameworks developed from the literature (60). Deductive codes are derived from existing literature and/or theoretical frameworks, including explicitly defined conceptual constructs (61). In contrast, inductive analysis involves systematic engagement with the data to generate codes, categories, patterns, and themes as they emerge from the dataset itself (62, 63). This approach has been shown to enhance the rigor of thematic analysis (64).

In this study, we employed hybrid thematic analysis by integrating deductive coding informed by established literature with inductive coding derived from the LLM-generated responses. In the first stage, open codes were generated through zero-shot prompting in ChatGPT as shown in Figure 2, representing a data-driven inductive coding process. In parallel, parent codes were derived deductively from the theoretical framework reported in the literature. Axial coding was subsequently applied to examine conceptual relationships between open codes and parent categories. The inductively generated codes and deductively derived categories were compared and integrated through an iterative refinement process. During this stage, codes were repeatedly reviewed, merged where conceptual overlap existed, and refined to improve clarity. Both coding schemes were standardized into a unified structured codebook to ensure terminological consistency and conceptual alignment.

The parent themes derived from the literature were categorized as Individual level, Social level, Food environment, and Lived environment. A final coding matrix was developed to systematically compare literature-derived parent codes and LLM-generated open codes across comparison types (exact match, more specific, or additional). For reliability enhancement, selective coding was employed whereby a single dominant parent theme was assigned to each quote based on the most salient underlying determinant. This procedure minimized overlap across categories and improved analytical clarity.

This coding approach has certain limitations, including potential subjectivity in code interpretation and the absence of multiple coders. The use of LLM-generated responses may also introduce biases related to prompt design and model behavior. Additionally, assigning a single dominant theme to each response may oversimplify complex insights, and iterative refinement may overlook less frequent but meaningful themes. These limitations are considered when interpreting the findings.

#### 3.2.2 Inter-rater reliability assessment

Inter-rater reliability was assessed using Cohen’s Kappa coefficient to evaluate agreement between literature-derived parent codes and LLM-generated open codes after standardization. It was calculated using the following formula.


Pe=∑(Pi×Qi)

Cohen’s Kappa (κ) = (Po​ − Pe)/​​ (1 − Pe)​

where Po is the observed agreement and Pe is the expected agreement.

O’Connor and Joffe (65), Cole (66), and Rau and Shih (67) highlight the importance of intercoder reliability measures, such as Cohen’s Kappa, in strengthening transparency, consistency, and methodological rigor in qualitative coding. Landis and Koch (67) proposed the following interpretation of Kappa values: ≤ 0 indicates no agreement; 0.01–0.20, slight agreement; 0.21–0.40, fair agreement; 0.41–0.60, moderate agreement; 0.61–0.80, substantial agreement; and 0.81–1.00, almost perfect agreement. These interpretive benchmarks have also been discussed and elaborated by McHugh (68).

## 4 Results

### 4.1 Characteristics of respondents (*n* = 914)

Primary data were collected from 914 undergraduate students enrolled in agriculture universities. The socio-personal profile is summarised in Table 1. The mean age of respondents was 20 years, and the average annual family income was INR 2,307,541. The mean nutrition literacy score was 20 (out of 32), while the mean Healthy Eating Behavior (HEB) score was 38 (out of 65). Male students constituted 58% of the sample. Regarding academic year, 33% were in the first year, followed by 31% in the third year and 25% in the fourth year. The majority of respondents identified as Hindu (87%). More than half belonged to socially and economically disadvantaged categories, including Other Backward Classes (OBC), Scheduled Castes (SC), Scheduled Tribes (ST), and Economically Weaker Sections (EWS). Approximately 57% of students were from nuclear families, and 44% reported parental education at the graduate level or above. Most respondents (72%) resided in college hostels. Based on Body Mass Index classification, 63% were within the normal weight range. In terms of self-perceived health status, 44% rated their health as fair and another 44% as good. With respect to nutrition exposure, 82.61% reported receiving nutrition-related information at college, and nearly half had completed nutrition-related coursework. Except for 12% of respondents, the majority expressed a perceived need for nutrition-related information.

**Table 1:** Socio-personal characteristics of the respondents (*n* = 914).

**Variable**	**Mean**	**Standard deviation**
Age (years)	20.42	2.12
Annual income (Rupees in lakhs)	23.08	0.04
Nutrition literacy	20.40	4.11
Healthy eating behavior	38.05	9.76

**Variable**	**Frequency**	**Percentage**
Gender		
Male	533	58.32
Female	381	41.68
Academic year (under graduation)		
First year	303	33.15
Second year	98	10.72
Third year	281	30.74
Fourth year	232	25.38
Religion		
Hindu	795	86.98
Muslim	53	5.8
Christian	43	4.7
Do not want to disclose	23	2.52
Social group		
General	218	23.85
Other backward classes (OBC)	458	50.11
Scheduled castes (SC)	127	13.89
Scheduled tribes (ST)	38	4.16
Economically weaker sections (EWS)	53	5.8
Prefer not to disclose	20	2.19
Family type		
Nuclear family	519	56.78
Joint	395	43.22
Parental education		
Lower primary	37	4.05
Upper primary	160	17.51
Secondary	137	14.99
Senior secondary	174	19.04
Graduation	289	31.62
Post-graduation	117	12.8
Place of stay		
College hostel	654	71.55
Rented accommodation outside campus	161	17.61
With family at Home	99	10.83
Body mass index category		
Underweight	223	24.40
Normal weight	574	62.80
Overweight	90	9.85
Obese	17	1.86
Data not available	10	1.09
Self perceived health status		
Good	403	44.09
Fair	398	43.54
Poor	113	12.36
Exposure to nutrition information		
Never	159	17.4
Seldom	140	15.32
Sometimes	397	43.44
Often	98	10.72
Always	120	13.13
Nutrition courses		
Have undergone	458	50.11
Not undergone	456	49.89
Self-perceived need for nutrition		
No need	110	12.04
Somewhat needed	343	37.53
Has a need	322	35.23
Has a great need	139	15.21

### 4.2 Comparing regression analysis results using LLM and statistical software

We employed ChatGPT to analyse the statistical relationships associated with healthy eating behavior using regression analysis on a dataset of 914 students. The LLM generated regression outputs in Excel format. The same dataset was independently analyzed using conventional statistical software (STATA). The results obtained from LLM were consistent with those provided by STATA software, as presented in Table 2. This demonstrated that the LLM-assisted analytical workflow can reproduce regression coefficients and statistical significance patterns comparable to those obtained from established statistical software. The variables Nutrition literacy, Place of stay, Self-perceived health status, Exposure to nutrition-related information and Self-perceived need for nutrition information were significantly associated with HEB among undergraduate students in both cases.

**Table 2:** Regression analysis using STATA and LLM for identifying predictor variables associated with HEB.

**Variables**	**STATA output**		**LLM Output**	
	**Coefficient**	***t-*value**	**Coefficient**	***t-*value**
Nutrition literacy	0.731***	9.45	0.731***	9.45
Annual income	0.001	−0.33	0.001	−0.33
Parental education (Base = Lower primary)				
Upper primary	−1.438	−0.89	−1.438	−0.89
Secondary	−1.441	−0.88	−1.441	−0.88
Senior Secondary	−1.106	−0.69	−1.106	−0.69
Graduation	−0.745	−0.48	−0.745	−0.48
Post graduation	1.261	0.75	1.261	0.75
Family type (Base = joint family)				
Nuclear family	−0.058	−0.09	−0.058	−0.09
Place of stay (Base = Rented accommodation outside campus)				
College hostel	−2.851***	−3.51	−2.851***	−3.51
With family at Home	1.002	0.87	1.002	0.87
Self-perceived health status (Base = Poor)				
Fair	1.725*	1.81	1.725*	1.81
Good	3.175***	3.26	3.175***	3.26
Exposure to nutrition related information (Base = Never)				
Seldom	−0.037	−0.04	−0.037	−0.04
Sometimes	1.189	1.41	1.189	1.41
Often	2.132*	1.85	2.132*	1.85
Always	2.664**	2.36	2.664**	2.36
Undergone Nutrition related courses (Base = Not undergone)				
Have undergone	0.656	1.08	0.656	1.08
Self-perceived need for nutrition information (Base = No need at all)				
Somewhat needed	0.028	0.03	0.028	0.03
Has a need	0.271	0.27	0.271	0.27
Has a great need	3.727***	3.28	3.727***	3.28
Constant	21.614	8.79	21.614	8.79

***p < 0.01, **p < 0.05, *p < 0.1.

Students with higher nutrition literacy exhibited better healthy eating behaviors (*p* < 0.001). Those residing in college hostels demonstrated significantly lower HEB compared to students living in rented accommodation outside the campus (*p* < 0.001). Students who reported fair health (*β* = 1.725, *p* < 0.05) and good health (*β* = 3.175, p < 0.001) demonstrated significantly higher levels of HEB compared to those reporting poor health. The magnitude of the coefficients suggests a graded positive relationship, with healthier self-perception corresponding to stronger healthy eating behavior. Compared to students who never received nutrition-related information, those who often (*p* = 0.064) and always (*p* = 0.018) received such information showed a statistically significant positive association with healthy eating behavior (HEB). Students who perceived a great need for nutrition information also demonstrated significantly higher HEB (*p* < 0.001). The model explains a modest proportion of the variation in HEB scores, with an *R*^2^ value of 0.2175, indicating that the included predictors capture part of the behavioral variation but that substantial unexplained heterogeneity remains.

### 4.3 Prediction of healthy eating behavior

#### 4.3.1 Experiment 1 (prediction without training data)

In this experiment, the dataset of 914 undergraduate students was provided to the LLM to predict HEB scores (range: 0 to 65) without using a separate training dataset. The model was instructed to use the provided predictor variables within a regression framework to generate predicted HEB scores. The predicted scores were compared with the observed respondent scores using a two-samples independent *t*-test (Table 3). The mean predicted HEB score was 35.644, whereas the mean observed score was 38.048. The t-test yielded a *p*-value <0.0001, which is below the significance level of *α* = 0.01; therefore, the null hypothesis of equal means was rejected. This indicates a statistically significant difference between predicted and observed scores, with the LLM systematically underestimating HEB. In all experiments, the HEB scores of the test datasets were withheld during prediction and used only for subsequent statistical comparison.

**Table 3:** Comparison of LLM-predicted data with real data in Experiment 1.

Variable	Observations	Obs. with missing data	Obs. without missing data	Minimum	Maximum	Mean	Std. deviation
Real score of HEB	914	0	914	13.000	65.000	38.048	9.762
LLM Predicted Score of HEB	914	0	914	30.347	65.000	35.644	2.497
Difference							2.404
Observed value of *t*							7.214
Critical value of *t*							1.961
DF							1826
*p*-value (Two-tailed)							<0.0001
Alpha							0.050

DF, Degrees of Freedom.

#### 4.3.2 Experiment 2 (train data: test data = 50:50)

The dataset was randomly divided into two equal subsets (*n* = 457 each). The first subset served as the training dataset, including HEB scores, and the second served as the test dataset, excluding HEB scores. The total HEB score (range: 0–65) was computed by summing item-level scores. The LLM was provided with predictor variables and coding descriptions from the training dataset and subsequently asked to generate HEB predictions for the test dataset. Predicted and observed scores were compared using a two-sample independent *t*-test (Table 4). The mean predicted HEB score was 37.458, compared to 39.155 for the observed data. The *t*-test yielded *p* < 0.001 (α = 0.05), leading to rejection of the null hypothesis. Predicted scores were significantly lower than observed scores.

**Table 4:** Comparison of LLM-predicted data with real data in Experiment 2.

**Variable**	**Observations**	**Minimum**	**Maximum**	**Mean**	**Std. deviation**
Real score of HEB	457	13.000	65.000	39.155	9.589
LLM Predicted Score of HEB	457	21.201	47.724	37.458	3.484
Difference					1.698
Observed value of t					3.557
Critical value of t					1.963
DF					912
*p*-value (Two-tailed)					<0.001
alpha					0.050

DF, Degrees of Freedom.

#### 4.3.3 Experiment 3 (train data: test data – 70:30)

The dataset was partitioned into 70% training data (*n* = 640) and 30% test data (*n* = 274). The LLM generated HEB predictions for the test dataset based on the training data. Comparison using a two-sample independent *t*-test (Table 5) showed a mean predicted score of 36.895 and a mean observed score of 41.080. The *p*-value (<0.0001) was below α = 0.05; therefore, the null hypothesis was rejected. Predicted scores were significantly lower than observed scores.

**Table 5:** Comparison of LLM-predicted data with real data in Experiment 3.

Variable	Observations	Obs. with missing data	Obs. without missing data	Minimum	Maximum	Mean	Std. deviation
Real score of HEB	274	0	274	13.000	65.000	41.080	9.955
LLM Predicted score of HEB	274	0	274	20.193	47.420	36.895	3.857
Difference							4.185
*t* (Observed value)							6.489
|*t*| (Critical value)							1.964
DF							546
*p*-value (Two-tailed)							<0.0001
Alpha							0.050

DF, Degrees of Freedom.

#### 4.3.4 Experiment 4 (train data: test data – 80:20)

The data were divided into 80% training (*n* = 731) and 20% test (*n* = 183) subsets. The LLM generated predictions for the test dataset. The mean predicted HEB score was 38.781, whereas the observed mean was 41.186 (Table 6). The *t*-test yielded *p* = 0.004 (<0.05), indicating a significant difference. Predicted scores remained significantly lower than observed scores.

**Table 6:** Comparison of LLM-predicted data with real data in Experiment 4.

**Variable**	**Observations**	**Obs. with missing data**	**Obs. without missing data**	**Minimum**	**Maximum**	**Mean**	**Std. deviation**
Real score of HEB	183	0	183	13.000	65.000	41.186	10.508
LLM Predicted score of HEB	183	0	183	22.543	47.153	38.781	3.545
Difference							2.404
Observed value of *t*							2.933
Critical value of *t*							1.967
DF							364
*p*-value (Two-tailed)							0.004
Alpha							0.050

DF, Degrees of Freedom.

#### 4.3.5 Experiment 5 (train data: test data – 90:10)

The dataset was split into 90% training data (*n* = 823) and 10% test data (*n* = 91). The mean predicted HEB score was 40.804, compared to an observed mean of 40.813 (Table 7). The *p*-value (0.994) exceeded α = 0.05; therefore, the null hypothesis was not rejected. No statistically significant difference was observed between predicted and actual scores, indicating convergence of predicted and observed means when a large proportion of the dataset was used for training.

**Table 7:** Comparison of LLM-predicted data with real data in Experiment 5.

Variable	Observations	Obs. with missing data	Obs. without missing data	Minimum	Maximum	Mean	Std. deviation
Real score of HEB	91	0	91	13.000	63.000	40.813	10.430
LLM Predicted score of HEB	91	0	91	23.420	50.500	40.804	4.950
Difference							0.010
Observed value of *t*							0.008
Critical value of *t*							1.973
DF							180
*p*-value (Two-tailed)							0.994
Alpha							0.050

DF, Degrees of Freedom.

#### 4.3.6 Experiment 6

Approximately 800 observations were used as training data, and 114 observations served as the test dataset. The LLM generated predictions for the test set. The mean predicted HEB score was 39.132, while the observed mean was 41.088 (Table 8). The *t*-test yielded *p* = 0.052 (>0.05); thus, the null hypothesis was not rejected. Predicted scores were statistically comparable to observed scores. These findings suggest improved predictive alignment as the proportion of training data increased, although this observation is specific to the present dataset and should not be generalized.

**Table 8:** Comparison of LLM-predicted data with real data in Experiment 6.

Variable	Observations	Obs. with missing data	Obs. without missing data	Minimum	Maximum	Mean	Std. deviation
Real score of HEB	114	0	114	13.000	63.000	41.088	10.060
LLM Predicted score of HEB	114	0	114	23.000	48.000	39.132	3.666
Difference							1.956
Observed value of *t*							1.951
Critical value of *t*							1.971
DF							226
*p*-value (Two-tailed)							0.052
Alpha							0.050

DF, Degrees of freedom.

To provide a more comprehensive evaluation of predictive performance, additional evaluation metrics- root mean squared error (RMSE), mean absolute error (MAE), and Pearson correlation coefficients were calculated (Table 9). These metrics quantify prediction error magnitude and the strength of association between predicted and observed scores. While R^2^ provides an indication of model fit within the regression framework, it does not directly reflect out-of-sample prediction accuracy. Therefore, error-based metrics (RMSE and MAE) are used to evaluate predictive performance as reported in Table 1. Prediction errors gradually decreased as the size of the training dataset increased, as reflected in declining RMSE and MAE values. Pearson correlation coefficients improved with larger training sets, indicating enhanced alignment between predicted and observed patterns. However, these should be interpreted cautiously, as they reflect the strength of linear association rather than the magnitude of prediction errors.

**Table 9:** Comparison of prediction performance between LLM-assisted workflow and baseline OLS regression model.

**Experiment**	**Training data (%)**		**RMSE**	**MAE**	**Pearson correlation (*r*)**
Experiment 1 (No training data)	0%	LLM	9.701	7.578	0.269
		OLS	8.620	6.640	0.466
Experiment 2	50%	LLM	8.897	6.879	0.414
		OLS	8.371	6.493	0.443
Experiment 3	70%	LLM	9.980	7.888	0.411
		OLS	8.109	6.394	0.429
Experiment 4	80%	LLM	9.539	7.556	0.501
		OLS	8.054	6.285	0.480
Experiment 5	90%	LLM	9.629	7.534	0.383
		OLS	7.944	6.052	0.436
Experiment 6	~88% (800 observations)	LLM	8.763	7.079	0.556
		OLS	7.760	5.980	0.450

Across all six experiments, the standard deviation (SD) of the observed healthy eating behavior (HEB) scores ranged from 9.59 to 10.51, whereas the SD of the LLM-predicted scores was substantially lower, ranging from 2.50 to 4.95. This marked reduction in dispersion indicates that the LLM consistently generated predictions that were tightly clustered around the mean and did not adequately reproduce the full variability observed in the real data. Such reduced dispersion is consistent with regression-to-the-mean effects commonly observed in predictive modelling (69), where predictions cluster closer to the average outcome and exhibit limited spread.

Similarly, the RMSE values for the LLM ranged from 8.76 to 9.98, which is large relative to the observed SD. This means that the average prediction error was nearly as large as the natural variation in HEB scores across students. Such a pattern suggests that the model captured general central tendencies but had limited ability to distinguish between individuals with substantially different eating behaviors. Although experiments with larger training datasets (Experiments 5 and 6) produced mean predictions that were not statistically different from observed means, the combination of compressed SD and relatively high RMSE indicates that predictive performance remained modest. Therefore, the LLM-assisted workflow appears useful for approximating aggregate patterns and average scores, but its utility for accurate individual-level prediction is limited.

Based on the comparison of predicted and observed values, including summary statistics and error metrics (RMSE and MAE), the pattern of prediction errors suggests that they are not purely random. Instead, the LLM predictions exhibit a systematic shrinkage effect, particularly evident in experiments with smaller training datasets, where higher values tend to be underestimated and lower values overestimated. This indicates a bias toward mean-centered predictions and suggests that, although aggregate trends may be approximated, the full heterogeneity present in the empirical dataset is not fully reproduced. Similar methodological concerns have been noted in prior evaluations of LLM-generated behavioral predictions (46, 70–75).

To benchmarck performance, a baseline ordinary least squares (OLS) regression model was estimated using the same predictor variables and training-test splits. The ordinary least squares (OLS) regression model was selected as the primary baseline due to its widespread use in behavioral and social science research, where interpretability, transparency, and ease of implementation are important considerations. OLS provides a standard reference framework for evaluating predictive performance in structured survey data and allows for direct comparison with conventional statistical approaches commonly used in this domain. However, the comparison remains limited to a single baseline model. The absence of additional machine learning models, such as random forest or gradient boosting approaches, represents a limitation of the present study. These models may be better suited to capturing non-linear relationships and complex interactions among variables. Future research could extend this analysis by incorporating such models to provide a more comprehensive benchmarking of LLM-assisted prediction.

As presented in Table 1, the OLS model produced slightly lower RMSE and MAE values across experiments, suggesting slightly better predictive accuracy compared to the LLM-assisted workflow. While the LLM approach was able to capture general patterns in the data, it did not outperform the conventional regression model. However, Pearson correlation coefficients between predicted and observed scores were broadly comparable between the two approaches, suggesting that the LLM captured similar underlying statistical relationships within the dataset. In both models, predictive performance improved as the size of training dataset increased. Overall, while conventional regression models demonstrated modestly stronger predictive precision, the LLM-assisted analytical workflow reproduced comparable prediction patterns when applied to structured behavioral survey data. The current contribution of the LLM lies more in facilitating analytical workflows through an interactive interface rather than improving predictive performance beyond established statistical methods.

### 4.4 Identifying determinants of healthy eating behavior using qualitative data

The determinants identified by the LLM from the selected quotes were classified as open codes, whereas those reported in the literature were designated as parent codes. These were compared using predefined comparison categories (exact match, more specific, or additional), and subsequently organized under standardized parent themes (Table 10). The comparative analysis demonstrates strong conceptual convergence between the literature-based framework and the LLM-generated coding structure, with differences primarily observed in granularity and contextual elaboration. Several direct correspondences were identified, including alignment between “Knowledge of dietary recommendations” and Knowledge and skills, “Taste preference” and Physiological preferences, “Peer influence” and Social networks, and “Cost and affordability” and Food price and diet affordability. In multiple instances, LLM-generated codes provided greater specificity within broader theoretical constructs. For example, “Emotional health, stress levels” refines the category of Psychology, while “Food shelf life, shopping frequency” operationalizes aspects of Food characteristics. Similarly, “Social and family influence, mixed messaging” extends the concept of Social networks by explicitly capturing contradictory behavioral signals.

**Table 10:** Final coding matrix.

S. No.	Quotes used regarding healthy eating behavior	Literature code/Parent category	LLM generated code/ Open coding	Parent themes	Comparison type	Explanation
1	“I knew we should eat 5 portions of fruit and vegetables a day, but I did not know that could help to prevent cancer...” (89)	Individual level knowledge and skills (Knowledge)	Knowledge of dietary recommendations (Knowledge)	Individual	Exact match	Direct alignment; both refer to awareness and information
2.	“One barrier for me is emotional health. Like if I’m feeling down or I’m stressed out, I eat crappy food and I prepare terrible food for my family.” (84)	Psychology (Psychology)	Emotional health, stress levels, motivation and wellbeing (Psychology)	Individual	More specific	LLM expands psychological dimension into measurable components
3.	“Oh yes to support their goals in the gym or in sport is the primary reason [to eat healthy] for a lot of my mates...” (85)	Beliefs and attitudes (Beliefs)	Fitness and Sports Performance, Peer Influence (Social network)	Individual	More specific	Adds context (fitness goals) to peer influence
4.	“I do not eat fruits and vegetables, I do not like how [they] taste so I just decide not to consume them.” (86)	Physiological preferences (Physiological)	Taste Preference, Personal Choice (Physiological)	Individual	More specific	Taste aligns directly; “personal choice” adds slight nuance
5.	“You just eat the food you were brought up with...” (87)	Habits (Habits)	Upbringing and Cultural Influence, Family and Tradition (Environment)	Social	More specific	Explains origin and formation of habits
6.	“But they’ll still hand her another plate of ribs. They’ll say she need(s) to lose weight, but at the same time they are still handing her more food.” (88)	Social level Social networks (Social network)	Social and Family Influence, Mixed Messaging (Contradictory actions) (Social network)	Social	More specific	Captures contradictory influences within networks
7.	“...in terms of society, at least this is how I see it, people live according to the opinions of others rather in terms of what they feel like doing or what is actually good for them. Thus (...) not being used to taking a piece of fruit may also be related to this: ‘It is pointless, people would make fun of me’...” (89)	Socio-cultural acceptability and expectations (Socio-cultural)	Social Influence and Peer Pressure, Self-Perception and Social Acceptance (Socio-cultural)	Social	More specific	Breaks down broader socio-cultural norms into components
8.	“There’s all this junk food, you know, jumpin’ out at you...the supermarket. Buy one, get one free, great big bags of chips...” (90)	Marketing and media (Marketing)	Food Availability and Marketing, Promotional Offers and Pricing (Marketing)	Social	More specific	Adds pricing strategies and exposure mechanisms
9.	“...cost-wise, I think it’s more effective to get a cheeseburger... You get more nutrition for the buck... It would not make sense to get a salad.” (91)	Food environment Food price and diet affordability (Affordability)	Cost and Affordability, Perceived Nutritional Value (Affordability)	Food environment	Additional	Cost matches; “perceived nutritional value” adds new evaluative dimension
10.	“I’m not normally a chocolate person...except if it’s right there in front of you.” (92)	Food availability (Availability)	Food Availability and Accessibility, Situational Eating Habits (Availability)	Food environment	Additional	Accessibility matches; situational habits extend scope
11.	“For me the hardest part is when we shop for 10 or 15 days, you know that fruits and vegetables do not last that long so we do not buy them.” (86)	Food characteristics (Characteristics)	Food Shelf Life, Shopping Frequency (Characteristics)	Food environment	More specific	Adds operational aspects of perishability
12.	“It’s convenient and fast to be unhealthy.” (93)	Lived environment Convenience and time (Convenience)	Convenience, Time Constraints (Convenience)	Lived environment	Exact match	Direct conceptual alignment
13.	“I go by bus [to shop], so it is whatever I can hold, which is not much.” (84)	Built and natural environments (Environment)	Transportation Limitations, Physical Convenience (Convenience)	Lived environment	More specific	Specifies environmental constraints (mobility/access)

Standardized codes are indicated in parentheses under the respective columns for parent categories and open codes. All quotes on healthy eating behavior were sourced from the review by (39).

Conversely, certain literature-derived categories demonstrated broader conceptual scope. For instance, Socio-cultural acceptability and expectations encompassed elements such as peer pressure and self-perception, while Built and natural environments subsumed transportation-related constraints. The LLM additionally introduced contextually enriched subdimensions, including “Perceived nutritional value” and “Situational eating habits,” which were not explicitly delineated in the original framework but conceptually complemented existing categories. No LLM-generated codes were deemed irrelevant or conceptually inconsistent; however, certain constructs (e.g., “Motivation and well-being”) exhibited thematic overlap across categories, reflecting multidimensionality rather than misclassification. Overall, the findings indicate that LLM-generated codes demonstrate substantial conceptual alignment with established theoretical categories while offering increased specificity and contextual elaboration.

#### 4.4.1 Interrater reliability assessment

The obtained Cohen’s Kappa value of 0.75 indicates substantial agreement according to the established benchmarks proposed by Landis and Koch (67), where values between 0.61 and 0.80 reflect substantial agreement. This level of agreement reflects consistent matching between literature-derived parent codes and LLM-generated open codes following standardization. The result supports the methodological robustness of the hybrid coding framework and suggests that, under zero-shot prompting conditions, the LLM was able to identify determinants of healthy eating behavior with strong agreement compared to established qualitative categories.

## 5 Discussion

In this study, we evaluated the predictive performance of LLM- assisted analytical workflow using both quantitative and qualitative approaches. The psychological construct ‘Healthy Eating Behavior (HEB)’ was treated as the outcome variable, and its probable determinants were identified using LLM. Quantitative data on HEB and its predictors were uploaded in the model, which was instructed to analyse statistical relationships using regression analysis. The findings of this study should be interpreted as an exploratory evaluation of an LLM-assisted workflow, rather than as evidence of causal inference or definitive predictive superiority. Accordingly, this work is positioned as an exploratory and methodological investigation, rather than a predictive benchmarking exercise. The primary objective is to examine the capabilities and behavior of large language models under specific experimental conditions, rather than to establish definitive performance comparisons. Establishing causality would require more rigorous methodological frameworks such as structural causal models, experimental designs, or explicit control of confounding variables, which were beyond the scope of this exploratory study. Unlike parameter-efficient fine-tuning approaches such as LoRA or supervised fine-tuning, which update model weights using task-specific datasets, this study used prompt-based analytical conditioning within the LLM-assisted analytical environment. Recent research (76) indicates that weight-level fine-tuning on behavioral datasets can further improve predictive accuracy and generalisability. The LLM-assisted workflow produced regression outputs consistent with those obtained from STATA, suggesting analytical alignment at the level of statistical estimation.

In the second stage, the dataset of 914 students was provided to the model to generate predicted HEB scores. When predictions were generated without a separate training-test split, substantial deviations from observed scores were observed. This aligns with the findings of Liu et al. (77), who report that LLMs often struggle to integrate statistical knowledge during structured reasoning tasks. Similarly, Yu et al. (5) noted that LLMs frequently rely on surface-level statistical association rather than structured reasoning. In contrast, Lippert et al. (26) demonstarted that GPT-4 achieved forecasting accuracy comparable to 119 human experts (correlation of 0.89 between predicted and observed outcomes, compared with 0.87 for experts and 0.07 for GPT-3.5).

Subsequent experiments introduced structure training and test splits. The training dataset was first used to analyse statistical relationships between variables, after which the model generated predicted HEB scores for the test observations. Prediction accuracy improved progressively as the size of the training dataset increased, with statistically similar mean scores observed when approximately 800 observations were used for training. This finding underscores the dependence of LLM-assisted prediction on adequate sample size within structured behavioral datasets. Recent studies (78) similarly report improved distributional alignment when language models are trained on substantially larger survey datasets, suggesting that the present dataset represents a small-scale exploratory case. The observation therefore reflects dataset-specific characteristics rather than a universal threshold.

Qualitative analysis further indicated that the LLM could identify plausible behavioral determinants from textual inputs, although these interpretations remain exploratory and should not be interpreted as causal explanations. Prior work shows that additional domain-specific adaptation and structured training can enhance performance in new tasks (79), suggesting potential avenues for methodological extension.

An important pattern observed in the prediction experiments was the lower variability of predicted scores relative to observed survey responses. While the empirical HEB scores exhibited substantial dispersion, the predicted values were more tightly clustered around the mean. This suggests that the model primarily captured systematic statistical relationships while smoothing individual-level heterogeneity. Such behavior is consistent with regression-based estimation of expected values and has been reported in prior evaluations of LLM-based behavioral prediction. The findings therefore indicate that LLM-assisted workflows may be more effective at approximating population-level tendencies than reproducing full individual-level variability. Comparison with the baseline OLS model showed that although prediction patterns were broadly similar, the conventional regression model generally achieved slightly lower prediction errors.

Further, the experiments were conducted using single-run executions, and result stability across multiple runs was not evaluated. Also, the findings are conditional on the specific analytical pathway generated in the present runs and may not fully capture variability across alternative prompts, runs, or modeling pathways. We also emphasize that a larger number of repeated runs across varying dataset sizes would be necessary to systematically assess stability, convergence, and robustness of the predictions. Given the non-deterministic nature of large language models, outputs may vary across different executions.

Another limitation concerns generalisability. The dataset consisted exclusively of undergraduate students from agricultural universities in India, representing a relatively homogeneous group in terms of age, education, and knowledge exposure. Dietary practices and behavioral determinants may differ across demographic, cultural, and geographic contexts. The Healthy Eating Behavior scale was applied within this specific student population, and its interpretation may vary elsewhere. Accordingly, the approximate training data requirement observed in this study should be regarded as specific to the present dataset rather than universally applicable. Prior research also emphasises that LLM-based behavioral simulations are sensitive to prompt design and conditioning details (80), underscoring the importance of transparent reporting. The findings should therefore not be generalised beyond comparable student populations. Large language models can generate plausible synthetic Human Computer Interaction (HCI) self-report data useful for ideation and pilot testing, but such data require validation with real participants and raise concerns about potential misuse in crowdsourced research (37). Future research should evaluate the model performance using datasets from diverse demographic groups, geographic regions and socio-cultural backgrounds.

A key limitation of this study relates to methodological transparency and reproducibility inherent to the use of a closed LLM environment. The analysis was conducted via the ChatGPT web interface, where the underlying execution environment (e.g., model configuration, runtime processes, and internal libraries) is not fully accessible or controllable. Although prompts and workflows were standardized, the absence of explicit code-level execution and the inability to document system-level details limit full reproducibility. The current study demonstrates a proof-of-concept application of LLM-based analysis, and that full computational reproducibility remains constrained by the nature of the platform. Future research should address these limitations by incorporating multi-run evaluation of the experiments and variability assessment across analytical pathways to evaluate robustness. Additionally the use of API-based implementations that allow greater control over the computational environment, and providing detailed execution logs or code-based workflows, could enhance transparency and reproducibility. Our findings suggest that LLM-assisted analytical workflows may support exploratory prediction tasks in behavioral research. In this dataset, predictions approached observed mean scores when a sufficiently large training subset was used. LLMs may contribute to social science research through synthetic data generation (81, 82) and qualitative theme identification (83). However, reliance on LLMs for predictive inference requires caution due to risks of error and misinterpretation (23). Previous applications of NLP methods such as topic modelling combined with predictive modelling in nutrition datasets (35, 36), highlight the broader potential of computational methods in behavioral nutrition research.

## 6 Future directions

Future research can further explore the application of LLM-assisted analytical workflows in behavioral nutrition research using larger and more diverse datasets. Evaluating the predictive performance of these approaches across different populations, socio-cultural contexts, and behavioral constructs would help determine their broader applicability and robustness. Replicating similar prediction experiments using datasets from other demographic groups and geographic regions would be particularly useful for assessing whether the patterns observed in this study hold in different contexts. Future studies may also compare LLM-assisted analytical workflows with conventional statistical and machine learning models, such as regression-based approaches and tree-based models, in order to better understand their relative strengths and limitations in behavioral prediction tasks. In addition, integrating structured survey data with qualitative data sources may further enhance the interpretability of behavioral patterns. Addressing reproducibility, transparent prompt reporting, and multi-metric evaluation frameworks will be important for strengthening methodological reliability. As these systems evolve, they may offer complementary analytical support for complex behavioral datasets in nutrition and social science research.

## 7 Conclusion

The results indicate that LLM-assisted predictions were statistically similar to observed mean HEB scores when a large proportion of the dataset was used for training. However, this observation is dataset-specific and should not be interpreted as establishing a general training data requirement. In the qualitative component, LLM identified plausible behavioral determinants from textual inputs, though these remain exploratory interpretations rather than validated causal mechanisms. This study provides initial insights into the application of LLM in behavioral nutrition research. However, further research is needed to address challenges such as generalisability and system integration. Besides, the findings should be interpreted with caution as the analysis primarily identified statistical associations rather than true causal relationships, and predictive accuracy was evaluated using mean comparisons and error metrics (RMSE and MAE). The reduced variability of predicted scores suggests limited sensitivity to individual-level heterogeneity. Moreover, model’s performance depended on explicit user prompting rather than autonomous methodological selection.

Despite these limitations, the findings indicate that LLM-assisted analytical workflows can support exploratory prediction and interpretation of behavioral survey data. However, conventional statistical models such as OLS regression continue to demonstrate slightly stronger predictive accuracy. Therefore, LLM-based approaches should currently be viewed as complementary analytical tools rather than replacements for established statistical methodologies. Future research using larger, diverse datasets, and rigorous evaluation frameworks will be critical for determining the reliability and broader applicability of LLM-assisted behavioral prediction in nutrition and social science research.

## Data Availability

The raw data supporting the conclusions of this article will be made available by the authors, without undue reservation.
