# Supplementary material for: Exploring the potential of large language models in nutrition behavior prediction: evidence from college students
Source: Front Nutr. 2026 Jun 22;13:1769064. doi: 10.3389/fnut.2026.1769064 (PMC13333345; doi:10.3389/fnut.2026.1769064)

Supporting Information for

**Exploring the Potential of Large Language Models in Nutrition Behaviour Prediction: Evidence from College Students**

**Prediction Experiments Workflow**:

Note: Experiment 6 was conducted using a different ChatGPT account to avoid memory bias from previous experiments.

**Prompts used for experiments**

1. **Train:Test- 50:50**


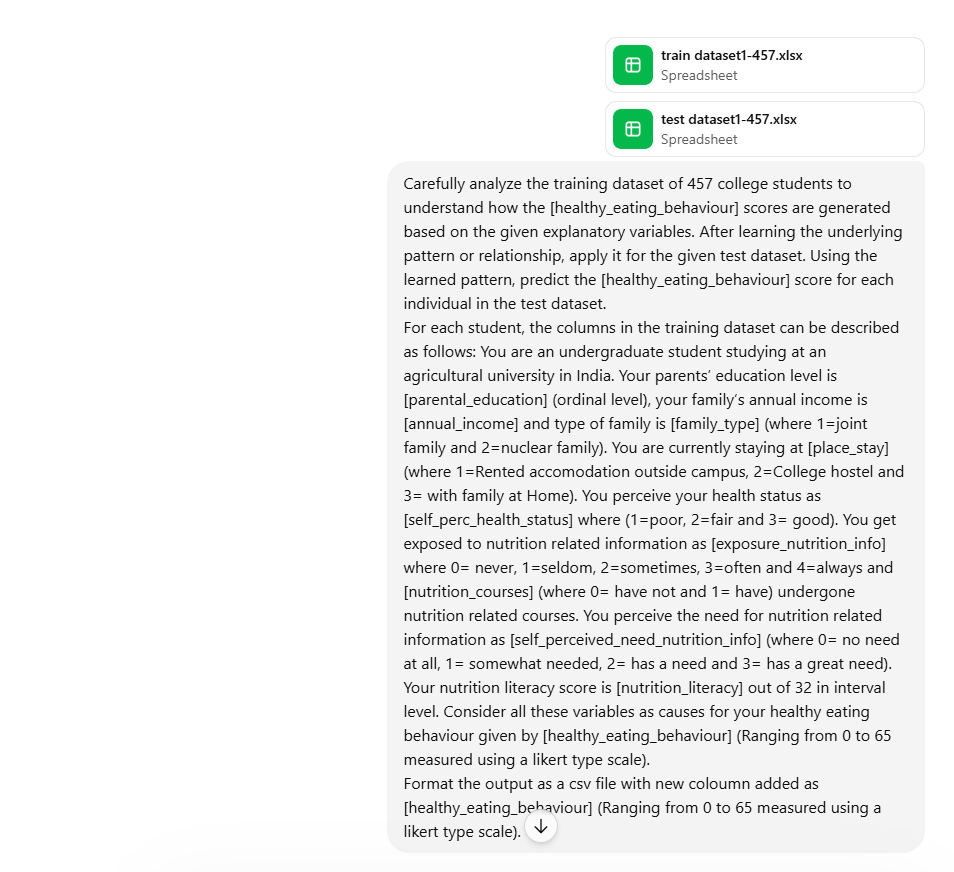


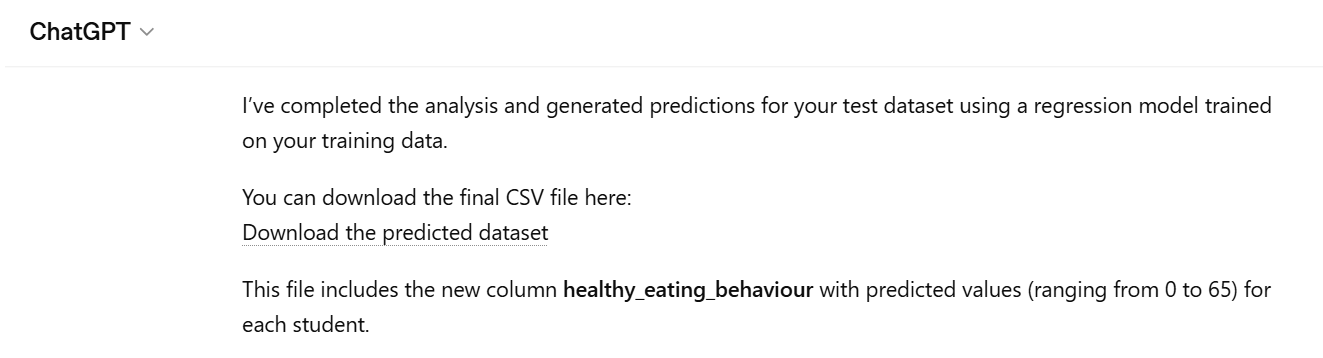


1. **Train:Test -70:30**


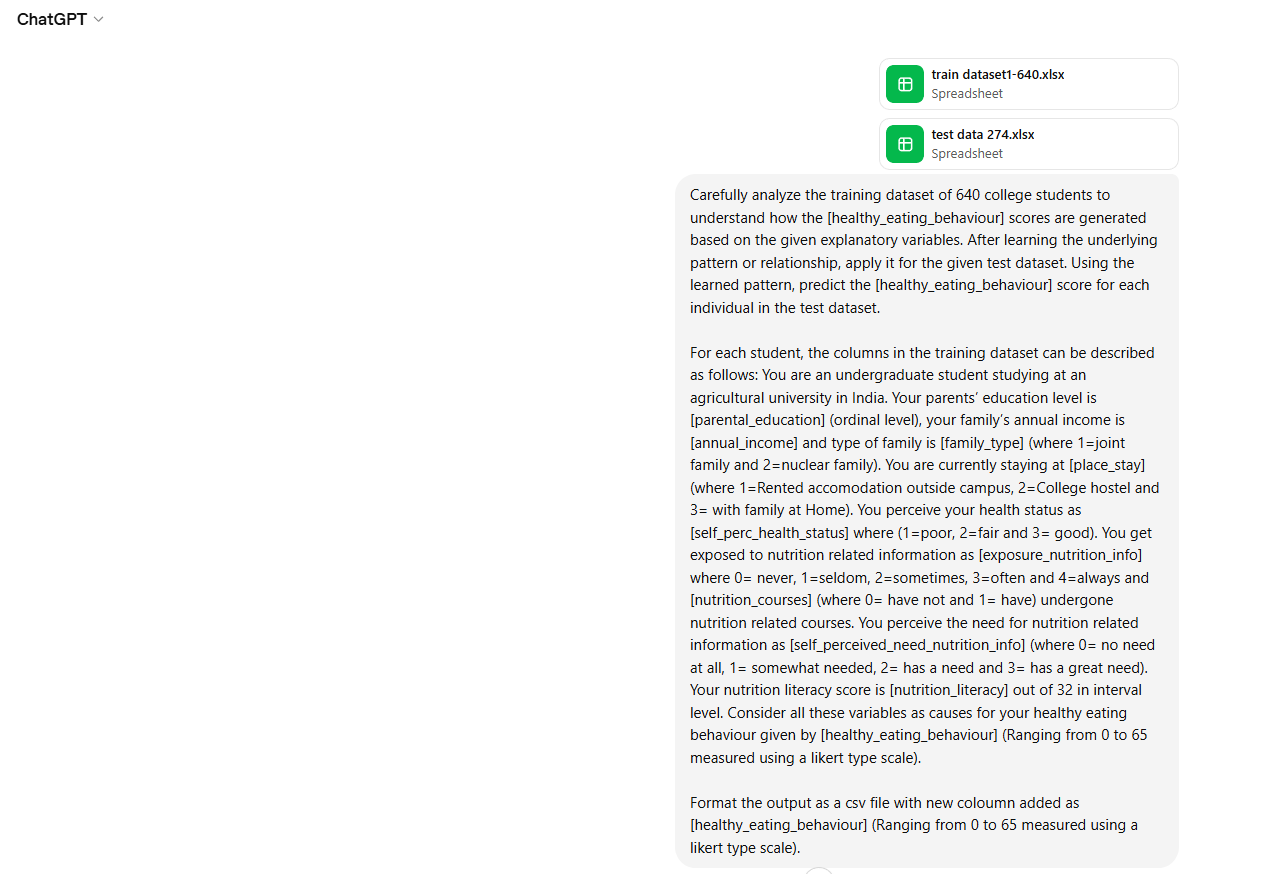


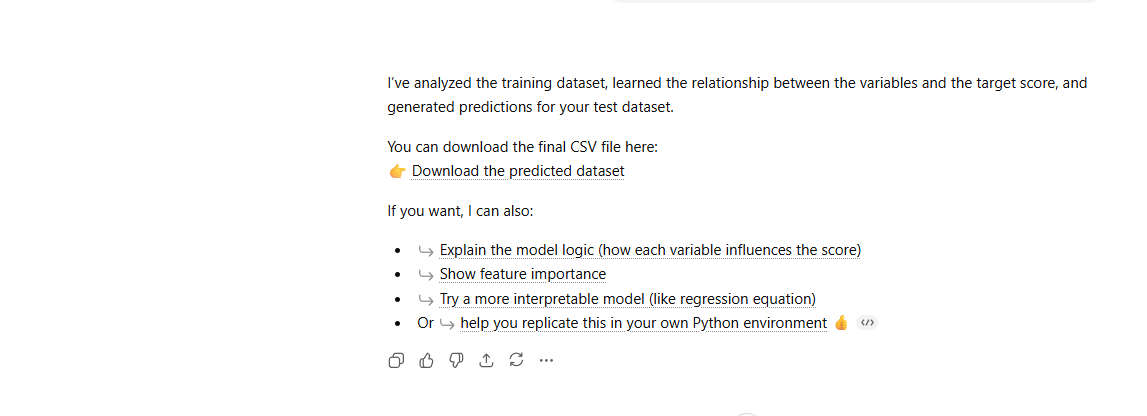


1. **Train:Test - 80:20**


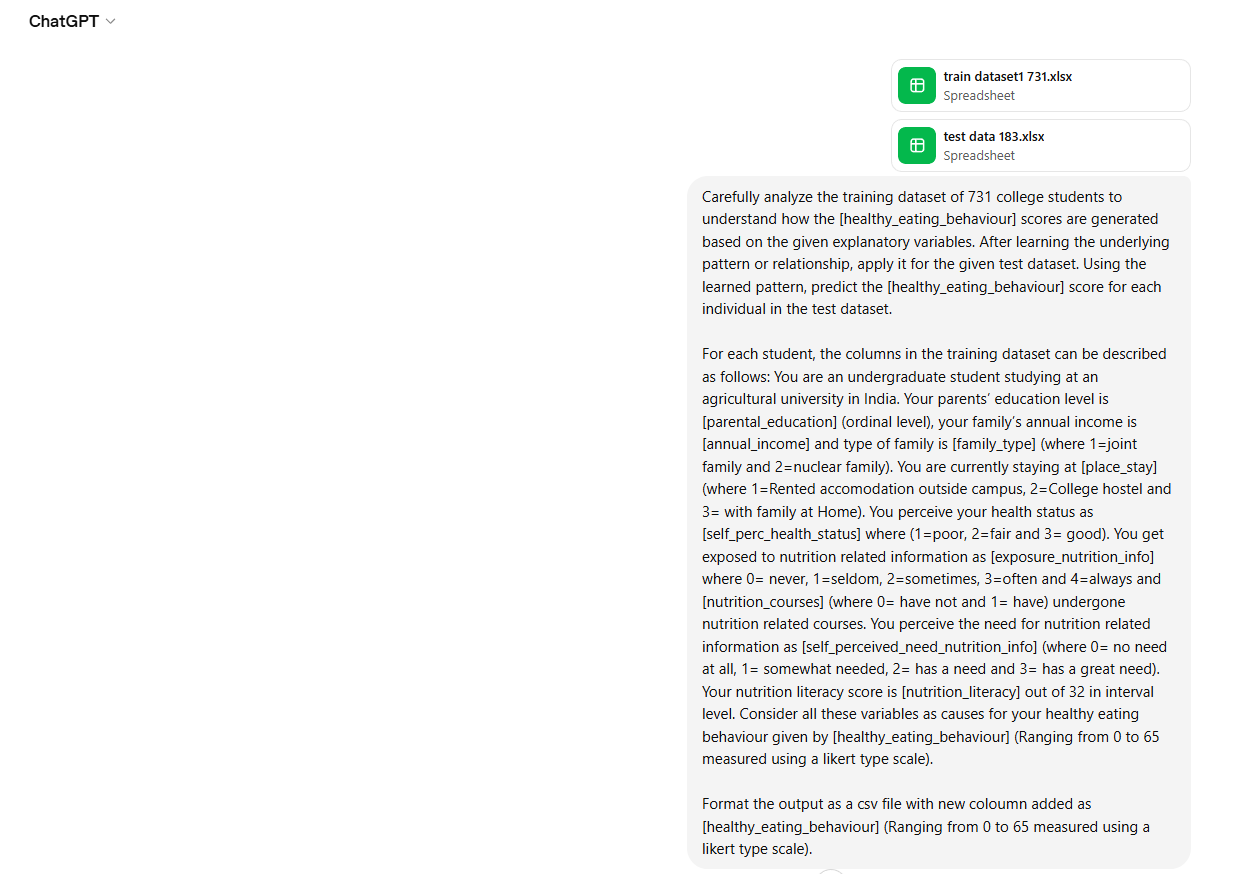


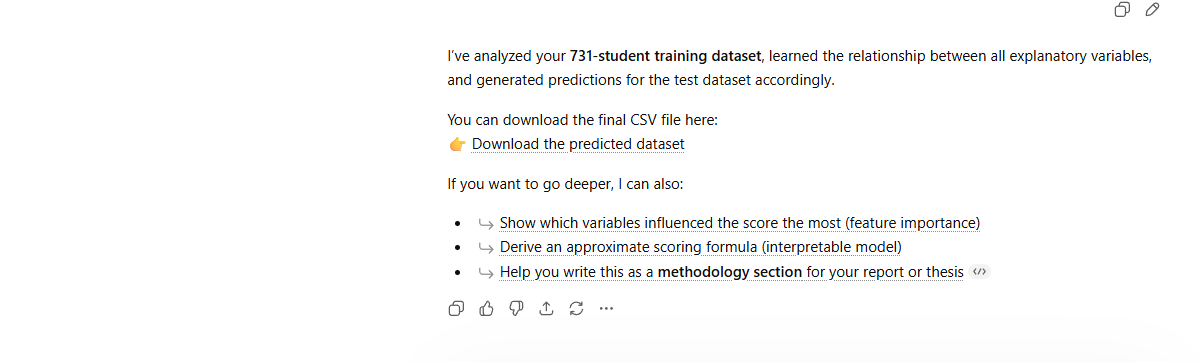


1. **Train:Test - 90:10**


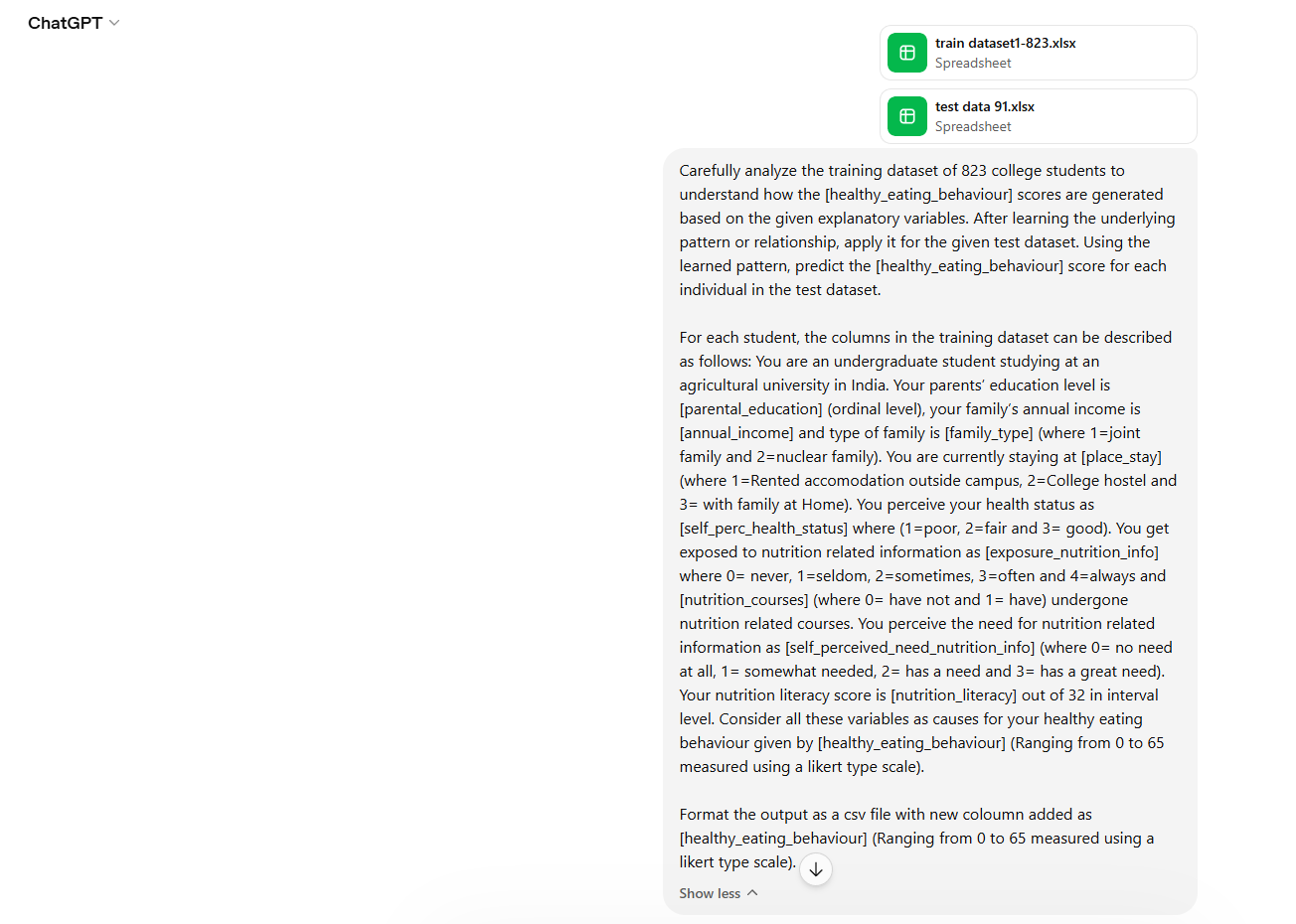


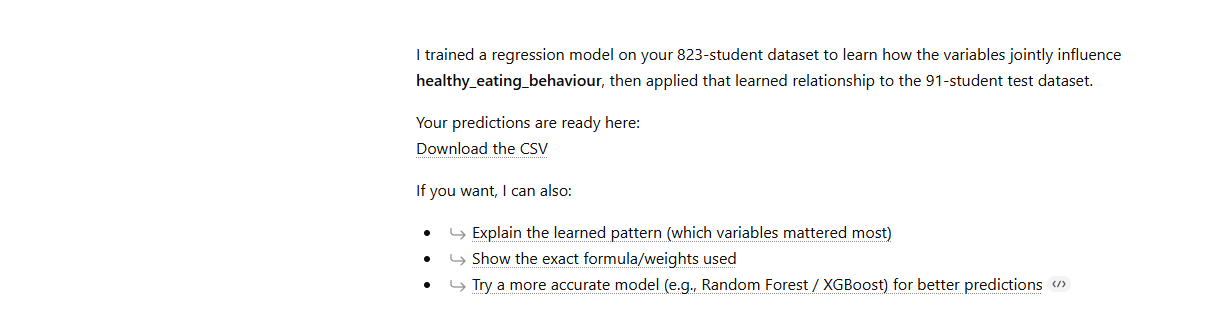


1. **Train:Test - 800 observations: 114 observations**


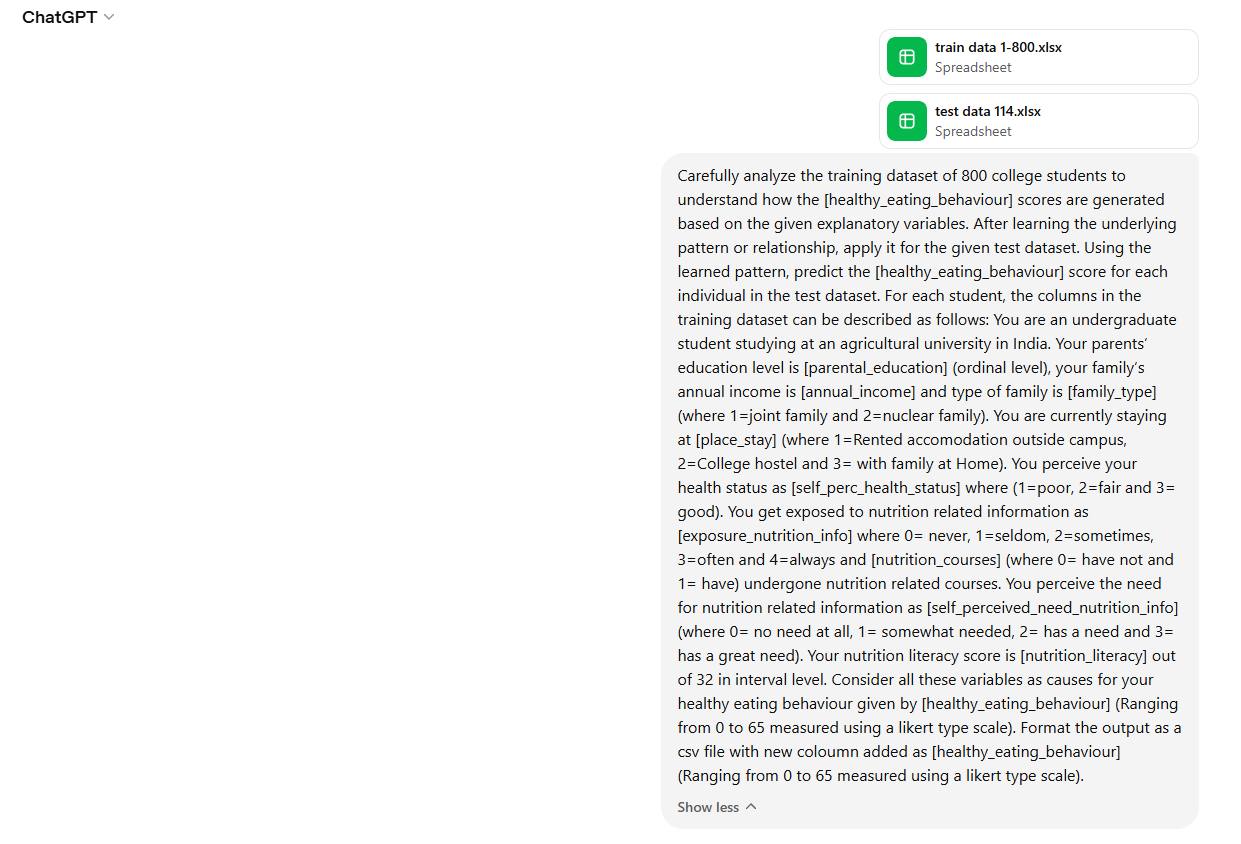


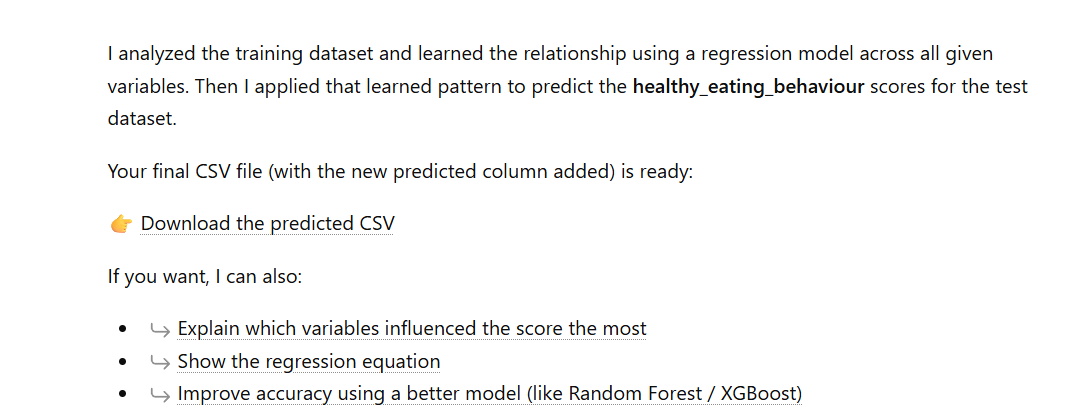

Supplement: Supplementary file 1 [file Table_1.docx]
